# Supplementary material for: Non-prescription acquisition of antibiotics: Prevalence, motives, pathways and explanatory factors in the Swedish population
Source: PLoS One. 2022 Sep 21;17(9):e0273117. doi: 10.1371/journal.pone.0273117 (PMC9491542; doi:10.1371/journal.pone.0273117)
Supplement: S1 Appendix — (PDF) [file pone.0273117.s001.pdf]

Appendix: questionnaire

1. How would you rate your general state of health?
    - 1 (Very poor) – 10 (Very good)

2. How much trust do you have in Swedish health care?
    - Very much trust
    - Fairly much trust
    - Neither much nor little trust
    - Fairly little trust
    - Very little trust

3. When was your last contact with healthcare regarding yourself?
    - Within the past week
    - Within the past month
    - Within the past six months
    - Within the past year
    - More than a year ago
    - Never

4. When was your last contact with healthcare regarding a child/family member?
    - Within the past week
    - Within the past month
    - Within the past six months
    - Within the past year
    - More than a year ago
    - Never

5. How many times during the past year have you been in contact with healthcare regarding yourself?
    - Once
    - Twice
    - 3-4 times
    - 5-6 times
    - 7 times or more

6. How many times during the past year have you been in contact with healthcare regarding a child/family member?
    - Once
    - Twice
    - 3-4 times
    - 5-6 times
    - 7 times or more

7. Have you used antibiotics (e.g., penicillin) prescribed by a doctor in Sweden in the last 12 months?
    - No

- Yes, once
- Yes, 2-3 times
- Yes, 4 times or more

8. How do you assess the veracity of the following statement: physicians in Sweden prescribe antibiotics too seldom

- 1 (Completely false) – 5 (Completely true)

9. In the last five years, have you obtained antibiotics without a prescription from a physician in Sweden?

- Yes
- No
- Don’t remember

Respondents stating “No” or “Don’t remember” proceeded directly to 13

10. In the last five years, how many times have you obtained antibiotics without a prescription from a physician in Sweden?

- Once
- Twice
- 3-4 times
- 5-6 times
- 7-8 times
- 8-10 times
- More than 10 times

11. How did you obtain antibiotics without a prescription from a physician in Sweden?

- I bought it at a pharmacy abroad without a prescription
- I bought it at a pharmacy abroad with a prescription from a physician there
- I received it from a family member or acquaintance
- I bought it from an online pharmacy without a prescription
- Other

12. Why did you obtain antibiotics without a prescription from a physician in Sweden?

- The physician refused to prescribe antibiotics
- I thought I would be denied a prescription if I went to a physician
- I wanted to keep antibiotics at home in case I would be denied a prescription
- It was easier than obtaining antibiotics via a prescription
- Other

13. If you or a close relative were to become ill at some point during the next five years, how likely is it that you would obtain antibiotics without a prescription from a physician in Sweden (regardless of reason)?

- Very likely
- Fairly likely
- Fairly unlikely
- Very unlikely

Respondents stating "Very likely" or "Fairly likely" proceeded to 14 and skipped 15. Other respondents proceeded directly to 15.

14. How important or unimportant are the following reasons why you would probably buy antibiotics without a prescription from a doctor in Sweden?
[Each rated from 1 (Very important) to 5 (Very unimportant)]

- Physicians don't want to prescribe antibiotics
- Avoid getting in contact with a physician
- I can decide for myself if I or a close relative/child need antibiotics
- I am abroad and can get antibiotics without a prescription
- I am abroad and can get antibiotics via prescription from a doctor there
- To keep antibiotics at home as a reserve in case I do not get it prescribed by physician
- Because it's easy

15. How important or unimportant are the following reasons why you would probably not buy antibiotics without a prescription from a doctor in Sweden?
[Each rated from 1 (Very important) to 5 (Very unimportant)]

- To avoid getting worse health
- In order not to contribute to antibiotic resistance
- The quality of over-the-counter antibiotics feels unsafe
- I trust that physicians can decide if I need antibiotics
- There is a risk of incorrect dosing (taking too much or too little of the antibiotic)
- Prescriptions are safer due to pharmacists' control
