# Supplementary material for: Non-prescription acquisition of antibiotics: Prevalence, motives, pathways and explanatory factors in the Swedish population
Source: PLoS One. 2022 Sep 21;17(9):e0273117. doi: 10.1371/journal.pone.0273117 (PMC9491542; doi:10.1371/journal.pone.0273117)
Supplement: Attachment — Submitted filename: Response to reviewers.docx [file pone.0273117.s002.docx]

**Response to reviewer comments**

We would like to extend our gratitude to all three reviewers for their detailed and helpful comments on our manuscript! We note the overall positive response to our reported study and the manuscript in general and welcome the many suggestions for improvement! We have done our best to adapt the manuscript accordingly (see manuscript file with tracked changes). Below follows a step-by-step account of how we have proceeded to revise the manuscript in light of the comments of specific reviewers. Note: when some comments were repeated several times in different places by a reviewer, we have chosen to cite the first mention by this reviewer in our description below.

**Reviewer #1:**

**This manuscript addresses the non-prescription use of antimicrobials in Sweden from the perspective of the issue of trust or lack of it in the healthcare system. Overall, a well conceptualized and written manuscript**

**Main concern is the use of mean as measure of central tendency with Likert-like responses such as Very much trust”, “Fairly much trust”, “Neither much nor little trust”, “Fairly little trust”, and “Very little trust. Using the median will be more appropriate in these cases. This will apply to Tables 1 and 2.**

Response:

We have revised the tables to include medians. We have also implemented changes in the text that describe tables 1 and 2.

**Table 3 and 4 are too bulky. The authors should kindly format according to the journal's specifications.**

Response:

To align with the journal's specifications, we have removed all the empty rows, thereby making tables 3 and 4 less bulky.

**Limitations: I am not sure if "limitations should come under "Results". The authors to kindly check the journal's template/ submission guidelines**

Response:

We agree that it is more natural to place “Limitations” under “Discussion” and have therefore moved this sub-section accordingly.

**Reviewer #2:**

**I think this paper has some interesting perspectives measuring the extent of acquiring antibiotics (AB) without prescription in a Northern European country. Even so, one would think there is no problem to explore, I think the authors do a good job in the introduction arguing for why such a study is still needed.**

**I think the authors fail really to discuss if the extend of acquiring AB as they measure it, proves if there really is a problem, that justifies spending time on the subject. In the introduction, the authors state p.5 that:**

**‘If a significant portion of people acquire antibiotics without prescription, the effectiveness of prescription control as a tool for AS would be undermined. Moreover, surveying prescription data to track the effect of AS on antibiotics use would provide a false picture that underestimates antibiotics use and overestimates the effectiveness of AS prescription control.’**

**However, I only see the authors making an indirect conclusion of whether they found a ‘significant portion’ of use of AB without prescription. Further, in the conclusion they don’t provide explicitly guidance to how the developed survey-tool could be used by authorities/ researchers from other countries:**

**‘The prevalence of non-prescription acquisition of antibiotics in the Swedish population is low, and the share of respondents intending to engage in this practice in the future is small. However, both past and intended non-prescription acquisition are strongly associated with low trust in health care. This highlights the importance of future studies of non-prescription antibiotics acquisition in low trust contexts and the need to consider trust preserving and enhancing measures when designing and implementing AS programs.’**

Response:

We understand the reviewer to expresses two distinct concerns here:

1) it is unclear that our findings warrant a conclusion as to whether “a significant proportion of people” engage in non-prescription acquisition of antibiotics.

2) there is insufficient explanation/discussion of how our survey tool could be used in other countries than Sweden.

In response to 1), we have clarified that our aim is not to determine whether “a significant portion of people” engage in non-prescription antibiotic acquisition but, more broadly, to clarify the extent of this phenomenon. Because this phenomenon potentially threatens the effectiveness of antibiotic stewardship and because almost nothing is known about its extent in Sweden (apart from a pilot study suggesting that it could be widespread), we think it is justified to spend time on the subject.

In response to 2), we agree that our specific survey tool may not be straightforwardly applicable in other contexts, and we have removed the sentence that suggested that it is from the introduction. In the Discussion, we now highlight the importance of studying the link between trust and non-prescription antibiotic acquisition in other contexts (in particular, low trust contexts), but we do not claim that such studies necessarily should apply our survey tool.

**The authors develop if, I understand correctly, a completely new survey, however, there is no description of relevance of hardly any of the specific content of the survey. This includes a lack of explanation of most of the included questions and pre-determined nominal answers. This is in particular a problem since some answers such as the one: ‘How important or unimportant are the following reasons why you would probably buy antibiotics without a prescription from a doctor in Sweden?, only contains answers, predetermined by the authors. Further, there is no explanation of any validation process of this new developed survey. And lastly, there is no precise explanation of the sampling procedure.**

Response:

We agree that the reasons for the design of the study were underdescribed. We have therefore amended the methods section significantly, describing in more detail how the construction of the survey was grounded in (1) an earlier pilot study which included qualitative elements, (2) related quantitative research by other groups on similar (though not identical) topics, and (3) general knowledge about factors that are known to tend to influence healthcare-seeking behavior. This account explains both our choice of having predetermined response options in the survey, which these are, and our choice of explanatory variables to test.

To describe the sampling procedure in more detail, we have added the following to the methods section:

“The Citizens panel consists of a pool of self-recruited respondents who answer questions about four times a year without remuneration. The respondents are invited to participate in each survey wave via email, and a maximum of three reminders are sent out during the field period. The respondents had to provide informed consent before they could take the survey. The number of respondents who did not consent and thus declined participation was 23 respondents (0,54 percent).”

**the key explanatory variable is ‘trust in health care’ or ‘institutional trust’. The authors explain that institutional trust can be measured with regard to trust in: government, agencies, or public institutions, and in relation to: if they are competent, have the motivation and the opportunity to fulfill their tasks in a good way. However, even so the authors describe the variable being multidimensional, only one question is asked in they survey: ‘How much trust do you have in Swedish health care?’ How do the authors know how respondents have interpreted this question, i.e. what their answers actually cover of all the possible dimensions. In addition, despite the multidimensional construct of ‘trust in health care’, there seems to be an understanding among the authors in the discussion that this question in the survey covers respondents’ trust in physicians, which I find is a huge possible misinterpretation.**

This is an important question, not at least in the light of the fact that trust in healthcare was found to be strongly correlated with the indicators of non-prescription antibiotic acquisition. In the design of the trust in health care question, we decided to use a uni-dimensional trust indicator. A systematic review of measures of trust in the health system in survey research found that most studies used a uni-dimensional indicator (Ozawa, S., & Sripad, P. (2013). How do you measure trust in the health system? A systematic review of the literature. Social Science & Medicine, 91, 10-14.). Trust in healthcare in Sweden has been measured using a uni-dimensional indicator since 1986 (<https://www.gu.se/sites/default/files/2021-04/9.%20Swedish%20trends%201986-2020_korrigerad%202021-04-26.pdf> page 11). Several studies already referenced have confirmed that responses to such questions are linked to behaviors and attitudes related to antibiotics (Eg.Carlsson, F., Jacobsson, G., Jagers, S. C., Lampi, E., Robertson, F., & Rönnerstrand, B. (2019). Who is willing to stay sick for the collective? – Individual characteristics, experience, and trust. SSM - Population Health, 100499. and Rönnerstrand, B., & Andersson Sundell, K. (2015). Trust, reciprocity and collective action to fight antibiotic resistance. An experimental approach. Social Science & Medicine, 142, 249-255.

However, we still agree that the multi-dimensionality of trust is important to consider in future studies of non-prescription use of antibiotics, not at least since trust seems to be an important factor to consider when it comes to non-prescription antibiotic use. So, instead of bringing up the dimensionality of trust briefly in the Introduction section, we have expanded this discussion and moved it to the Discussion section

We have thus added the following text (with additional references x1 and x2 – see below) to the Discussion:

“While the current study follows much of the previous literature and makes use of a uni-dimensional indicator of trust in the health care system (X1), theoretical arguments speak in favor of institutional trust being a multidimensional concept. Institutions can be trusted if they are competent, have the motivation and the opportunity to fulfill their tasks in a good way [X2]. Given the results of the current study, an interesting avenue for future research could be to measure the dimensionality of trust in the health care system and analyze what specific components of trust that may be important in curbing the demand for non-prescription antibiotics. One such factor could be the degree of trust in the competence characterizing the healthcare system or healthcare personnel. The perceived competence is likely to influence the degree to which patients think doctors' decisions not to prescribe antibiotics is reasonable and medically motivated, and this trust dimension may thus lower the demand for antibiotics without a prescription. Another trust component is the perception that an actor or institution has good intentions. This dimension of trust could potentially trigger a reciprocal and prosocial feeling of collective responsibility for the problem of antibiotic resistance. This way, people might want to contribute to the fight against antibiotic resistance.”

Added new references:

X1

Ozawa, S., & Sripad, P. (2013). How do you measure trust in the health system? A systematic review of the literature. Social Science & Medicine, 91, 10-14.

X2

de Fine Licht K, Brülde B. On Defining “Reliance” and “Trust”: Purposes, Conditions of Adequacy, and New Definitions. Philosophia. 2021;49,1981–2001

**there is no description of how the end responders represent or do not represent the Swedish population**

Response:

We agree that this is an important point warranting more extended comment. In the Result section we have therefore added the following section to describe the degree of correspondence between the respondents of the survey and the Swedish population 18+ (in 2020):

“Among the respondents who completed the survey, the gender balance was very equal – 51 percent were women and 49 percent men. The share of respondents who finished the survey being 70 years or older was 17 percent and the share of respondents with three years of university education or more was 27 percent. This is reasonably similar to the Swedish population over 18 years (2020), where the 70+ group was 13 percent of the population and the share of people with university education (three years or more) was 24 percent.”

**Reviewer #3**

**The manuscript is interesting, especially given the very low overall history and intent of receiving non-prescription antibiotics. The authors usefully frame their results as an example of what is possible in a high-trust environment. I especially appreciated the sentence “Merely denying patients antibiotics is not enough; rather these people need to be made sufficiently motivated not to acquire antibiotics from other sources.”**

**the wording of the main question in English is unclear. When I read the phrase “acquired antibiotics without a prescription from a Swedish physician” I took it to mean that Swedes were getting antibiotics directly from Swedish physicians, but without a prescription. Perhaps Swedish physicians were giving out sample antibiotics or had some other mechanism to give out antibiotics without issuing a prescription. (In a totally unscientific test, I asked my spouse how they interpreted the question, and they interpreted it the same way.)**

**It was not until page 11, at the beginning of the Results that includes the ways in which Swedes obtained non-physician-issued antibiotics that I understood the authors meant “acquired antibiotics, but not from a Swedish physician having issued a prescription.” Another way to word this in the Abstract and early in the manuscript is “acquired antibiotics, other than from a Swedish physician issuing a prescription.” With that, the authors’ meaning should be clear.**

Response:

We thank the reviewer for calling our attention to this ambiguity, which is due to translation of from Swedish to English. We now use the wordings suggested by the reviewer (or very similar ones) throughout the paper. For the sake of ease of expression, we sometimes use the phrase “non-prescription acquisition of antibiotics”, which we now define in the introduction as “acquiring antibiotics in other ways than from a Swedish physician having issued a prescription”. This should help prevent misunderstanding. However, we have not changed the wording of the survey questions as such (see Appendix) in order to keep as close as possible to the original Swedish wording. The question in Swedish does not suffer from the ambiguity pointed out by the reviewer, so it should not affect the reliability of our results.

**the authors repeatedly state that the sample is representative, but the respondents are non-representative for at least two reasons. First, to even be included in the survey, potentially participants had to give informed consent. Second, the sample was “pre-stratified to mirror the Swedish population,” but there was a 57% response rate. Those willing to give informed consent and respondents are likely to be systematically different from Swedes who did or would not give informed consent and non-respondents (e.g., in their interest in the topic, willingness to report non-socially acceptable or illegal behavior, and other factors). I would guess consenting respondents would be less likely to acquire non-prescription antibiotics than the average person. Do the authors have any data about how respondents were demographically similar or different from the Swedish population?**

Response:

The respondents had to give informed consent, but the number for respondents who did not consent was very small, in fact only 23 respondents (8 women and 15 men).

The total sample was pre-stratified to mirror the Swedish population in terms of age, education and gender. But the important question raised by the reviewer concerns the representativeness given the 57 percent participation rate.

Among the respondents who completed the survey, the gender balance was very equal – 51 percent were women and 49 percent men. The share of respondents who finished the survey being 70 years or older was 17 percent and the share of respondents with three years of university education or more was 27 percent. This is reasonably similar to the Swedish population over 18 years (2020), where the 70+ group was 13 percent of the population and the share of people with university education (three years or more) was 24 percent.

Even though the sample is reasonably similar to the Swedish population in terms of gender, age and education, we agree with the reviewer that the issue of representativeness needs to be discussed with somewhat more caution, one reason being that we cannot rule out that there might be other variables that would improve the estimates of behaviors and attitudes towards antibiotics, which were not included in the pre-stratification process.

For this reason, we have changed some wordings regarding representatives (Abstract and Introduction) and added the following text in the limitation section.

“The results reported in this paper come from an online survey pre-stratified to mirror the Swedish population in terms of age, gender and education, and the sample is reasonably similar to the Swedish population with regard to these factors. However, one limitation in the data is that there might be other variables that would improve the estimates of behaviors and attitudes towards antibiotics, which were not included in the pre-stratification process.”

**I would want the authors to report on the overlap of respondents who said they had obtained non-prescription antibiotics in the past and intend to do so in the future. Are these the same people? If so, this problem could be very limited. If not, the disconnect between past behavior and future intended behavior could be interesting for the authors to discuss.**

Response:

Thank you for this interesting suggestion, we agree that it is interesting to report on this overlap. We have added the following text to the result section.

“The likelihood of future acquisition of antibiotics without prescription differ depending upon previous non-prescription antibiotic acquisition. Among the respondents who previously obtained antibiotics without a prescription from a physician in Sweden, about 30 percent reported that they are likely to obtain antibiotics without prescription in the future (30 out of 97 respondents). This share is less than 4 percent among respondents who responded not having obtained antibiotics without a prescription from a physician in Sweden (153 out of 4081).”

**the authors never state what kind of statistical analysis they are using. Also, it is not clear how the authors are evaluating each variable in a model. I would think they should be using a Type 3 test or a test-of-trend for some of the variables, but there appear to be p-values for all levels of all variables.**

Response:

We have now added a section called Statistics (under methods):

“Statistics

Two multiple logistic regression models were used to investigate the association between the explanatory variables and the two dependent variables: 1) previous acquisition of antibiotics without a prescription (Tabel 3) and 2) likelihood of future acquisition of antibiotics without a prescription (Tabel 4). Table 3 and 4 reports Odds ratios (ORs) and 95% confidence intervals (CIs). All data were analysed with the STATA 17 statistical software package. “

**the data presented in the tables is difficult to interpret. The authors do not describe how importance was asked in the Methods section.**

Response:

The question wording is given in the table comments. But for clarity, in the revised version of the manuscript, we now also report this in the text.

**the Ns are hard to follow. For Table 1, each variable has an N of about 160 to 170, but 183 respondents said they were somewhat or very likely to obtain antibiotics in the future. Are these the same subsets? The tabular results need to more clearly state who is responding to each question.**

We have added the following text for greater clarity:

“This question was only asked to respondents who in the previous question stated that it is “Somewhat likely” or “Very likely” that they will obtain antibiotics without a prescription in the future (n=183). The number of respondents who responded to the questions about reasons for obtaining antibiotics without a prescription in the future varied between 161-167.”

**Page 4, 1st paragraph: The Introduction seems like a repeat of information that should be in an Abstract rather than an introduction.**

Response:

We have removed the first paragraph of the Introduction, which indeed repeated information also found in the abstract.

**Page 4-9: The Background and Introduction are very long. I am accustomed to a Background section that is 1-2 pages and gets right to the point of the study.**

Response:

We have merged the Introduction and Background sections into one, and significantly shortened it. As mentioned above, the first paragraph of the main text has been removed. In addition, the passages on trust have been shortened and more clearly integrated in the argument that motivates our study. Also, several other sentences in the Introduction have been moved or trimmed to make the presentation more concise.

**Page 9, paragraph 3: The authors need to explain the parenthetical “AAPOR RR5” and why they chose that method of calculating the response rate. Also, the reporting of responses and response rate seems like it belongs in the Results section.**

Response:

We have deleted the parenthetical AAPOR RR5 and instead explain the method for calculating the participation rate. Under the RESULT SECTION, we have added the following text:

**“**The survey yielded 4243 responses and the participation rate was 57 %. We calculated the participation rate by the quotient of the number of respondents who completed more than 50 percent of all questions in the survey and the number of respondents in the initial sample.”

**Page 12, Table 1: The authors should clarify in the table title whether this refers to past behavior or future intent (I believe it is future intent).**

Response:

It has now been clarified that Table 1 reports future intent.

**Page 15, Table 3: There are some formatting problems with some of the confidence intervals. Perhaps the hyphen is missing?**

Response:

This formatting problem has now been resolved.

**Page 17, paragraph 2: The authors are incorrect in saying the odds ratio represents something is “9 times higher.” It is actually “9 times the odds,” which is different.**

Response:

This mistake has been corrected.

**Page 20, paragraph 1: The authors should include data to their statement that Swedes have a “high level of awareness about antibiotic resistance.”**

Response:

Two references have been added to support this statement.

Grigoryan, L., Burgerhof, J. G. M., Degener, J. E., Deschepper, R., Lundborg, C. S., Monnet, D. L., . . . Haaijer-Ruskamp, F. M. (2007). Attitudes, beliefs and knowledge concerning antibiotic use and self-medication: a comparative European study. Pharmacoepidemiology and Drug Safety, 16(11), 1234-1243. doi:10.1002/pds.1479

European Commission (2018). *Special Eurobarometer 478 Antimicrobial Resistance Report – Wave EB90.1*. Brussels: Kantar Public. Avaliable online: <https://www.eusaferhealthcare.eu/wp-content/uploads/ebs_478_en-1-min.pdf>

**Figure: There is no key for the y-axis. I presume this is %.**

Response:

A key for the y-axis has been added.
